# COMPUTER SIMULATIONS IN SERVICE OF BIOLOGY

**DOI:** 10.3389/frym.2020.603629

**Published:** 2021-09-07

**Authors:** Michael Levitt

**Affiliations:** Department of Structural Biology, Stanford University School of Medicine, Stanford, CA, United States

## Abstract

Computer simulation is an important research tool in today’s scientific world. Computers allow us to perform computations that mimic the behavior of complex (biological) systems in ways that we could not otherwise achieve. You could think of these simulations as a computer game, in which a virtual world is created that works according to certain (e.g., physical) rules. While we play the game, we learn the rules governing this virtual world and its environment, and also the way that we affect this world as players. In this article, I will explain how we use computer simulations in the world of structural biology to study the structure and function of molecules. I will also describe how I think that we could use insights from the world of biology and computer simulations to advance the society that we live in.

## Graphical Abstract

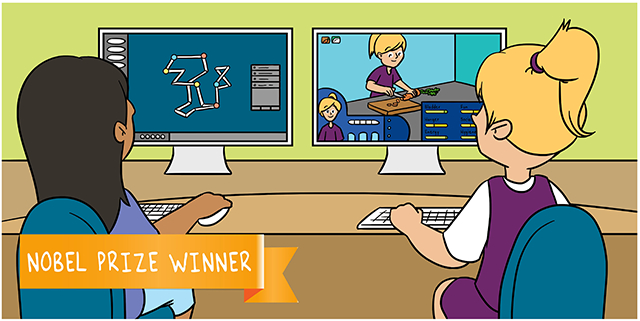

Professor Levitt won the Nobel prize in Chemistry in 2013 for the development of multiscale models for complex chemical systems

## WHAT IS A COMPUTER SIMULATION?

**Figure 1:**
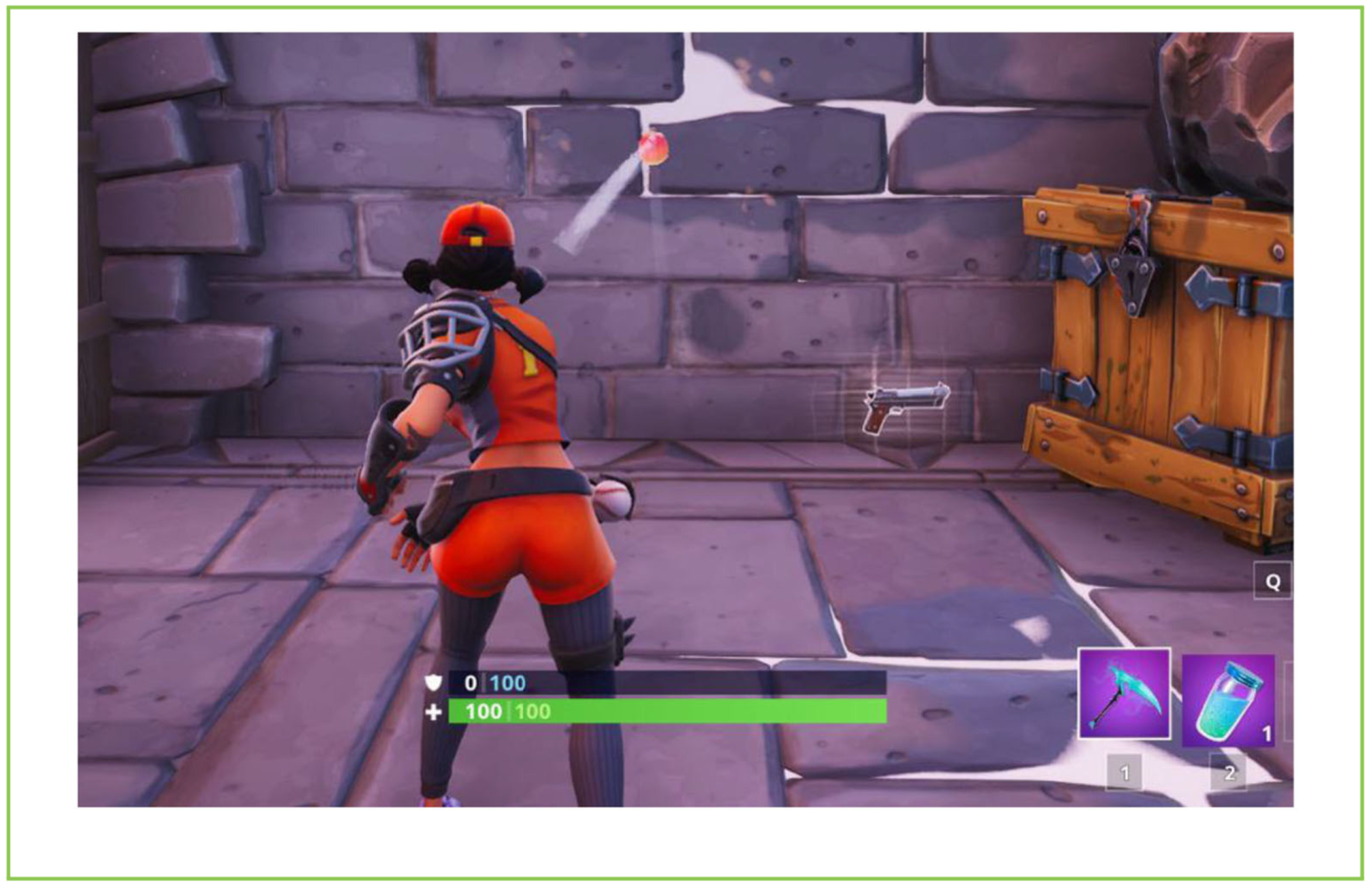
An example of a computer simulation as implemented in computer games. The figure is taken from the online game “Fortnite.” The challenge is to throw the ball so that it hits the objects in the room 15 times before it falls to the ground. This game is actually a simulation of physical laws. To realistically present the motion of the ball in the game visually, the computer needs to compute the physical path of the ball based on physical equations—Newton’s equations of motion. In other words, the computer simulates physical laws and presents the result on the screen. By adopting the same principle, the computer can simulate different processes in nature, such as weather or biological processes, to help us better understand them (Source: Forbes).

A simple way to understand a **computer simulation** is to think about computer games. Think, for example, about an adventure game, in which your character walks around an environment performing various actions. For the game to look realistic, the computer must build a virtual world that behaves like the real world. For example, if you throw a ball during the game, the computer must use the appropriate physical equation (Newton’s equation of motion, in this case) in order to compute the motion of the ball and create a realistic simulation of the physical path of the ball during its motion (Figure 1). By the same principle, the computer can simulate other real-life processes, assuming we know the physical laws that govern them. In other words, not only the laws governing the motion of objects could be simulated, as we saw in the above example, but also more complex processes, such as the weather, chemical reactions, and also a variety of biological processes, such as the folding of **proteins**, which we will discuss below.

## WHAT CAN WE LEARN FROM COMPUTER SIMULATIONS?

Let us think about an adventure game, like Assassin’s Creed. Assuming that your mission takes place in Florence, Italy, you walk around this city using a computer simulation of the streets of Florence. While walking, you see various houses and historical sites, such as the beautiful cathedral called the Duomo. After many hours playing this game, you will know a great deal about the geography of Florence. With this knowledge, you could walk around the real city of Florence, feel familiar with it, and identify different places and sites that you encountered in the computer game. This means that the game provided you with real knowledge of the city itself, even though you had never actually visited the city. Such learning through computer simulations is a safe process—you are not afraid to get hurt while playing the game, so you can let yourselves perform actions in the game that you would not dare to perform in real life. Depending on the game, sometimes there are even actions that cannot be performed in real life but can be done in the computer game (like you having the power to fly or meeting imaginary creatures).

This principle of acquiring knowledge through computer simulations is also used in the scientific world: we build a model of a physical or a chemical process that we are studying and then simulate it using a computer. The model is based on mathematical equations describing the process (as in Figure 1, where the model utilizes the equations that describe Newton’s laws and govern the physical behavior of a ball). The computer allows us to view how the process unfolds with time, so we can examine whether the results of the simulation fit the real-world process. If the results fit, then we conclude that the model is good and could be used to better understand the phenomenon that we are investigating. If the computer results do not fit the real-world results, then we conclude that the model needs to be revised. Modifying the model can help us to identify errors in our understanding of the process we are studying. Since a simulation is not dangerous, we might try all sorts of models and possibilities that might even be impossible to explore in real life situations. Sometimes there are surprises during such computer simulations, and we might find that a “wild” model that we have examined actually best describes the phenomenon under investigation. Computer models give us the freedom to be creative and find explanations of reality that are otherwise hard to find.

## JUST RIGHT

**Figure 2:**
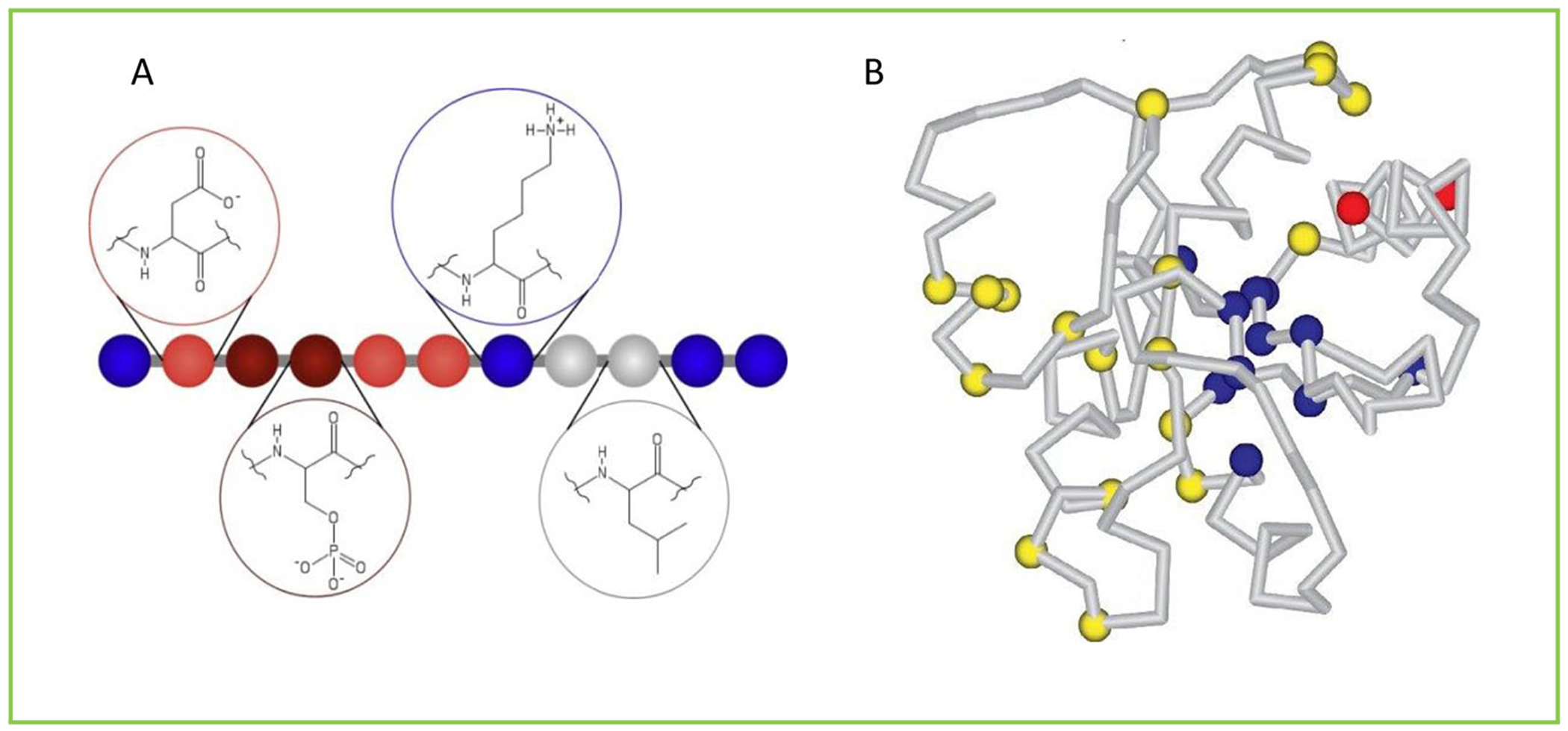
The folding of proteins. **(A)** A simple model for simulating a protein using a computer. The protein is described as a necklace composed of beads with different features. Each color describes a different type of bead, and each bead describes a collection of atoms and their interactions (as you can see in the circles above or below the beads) (Adopted from Cragnell et al. [2]). **(B)** A simple model of a protein as a necklace of beads that also includes the mathematical equations describing the interactions between beads of specific colors. This model is sufficient to describe the folding of proteins into stable 3D shapes (Adopted from Researchgate).

When using computer simulations in science, one of the most important principles is one that I call “just right.” According to this principle, we need to build a model that is not too simple and not too complicated. If the model is too simple, it will not describe the phenomenon we want to investigate in sufficient detail. In contrast, if the model is too complicated, we will not be able to use it to get information that will contribute to our understanding. I think that every researcher should understand what they are doing at a simple and basic level, so that they can explain their research to others. If someone says that they have discovered something great but it is too complicated to explain, I get filled with doubts and I am not convinced that they really understand what they are studying. Therefore, I always search for the simplest model that is good enough (as you will see in Figure 2 below about the folding of proteins). I believe that this is a very general idea for life—each explanation has its own “just right” level. Therefore, I advise you to always look for the simplest explanation that clarifies what you are trying to understand—not more and not less.

## COMPUTER SIMULATIONS IN STRUCTURAL BIOLOGY

I will now show you how we used computer simulations and the “just right” principle to understand a very important phenomenon in biology—the folding of proteins. Research into the structure of proteins is part of a field called **structural biology**. Let us think about how living organisms function. Inside the body, there are many string-like structures called proteins. These proteins fold up to create three-dimensional shapes. Each protein has its own unique shape, which is identical inside every living body. The amazing thing is that these 3D-shaped proteins perform all the functions of life—building the body, undergoing chemical reactions, moving the muscles, and digesting food. Therefore, understanding the process of folding that determines the final shape of a protein is extremely important.

Proteins are large molecules made from thousands of atoms with many interactions between them. If we want to run a computer simulation to deal with all these atoms and their interactions inside a protein, it becomes far too complicated. In the early 1970’s I worked on this problem with Arieh Warshel, and in 1975, we published our findings in an important scientific journal [1]. We found that we could build a simple model of a protein presented as a necklace composed of different types of beads, in which each type of bead has somewhat different features than those of other beads (Figure 2A). Each bead represents a collection of (say 10) atoms and their interactions. Specific beads (say red beads) are attracted to other specific beads (say blue beads). This simple model managed to provide an adequate and useful explanation for the folding of proteins (Figure 2B) and it has been accepted as a model for many other molecular computations [2]. These simulations allow us to understand, and even predict, the three-dimensional structure of different proteins and to better understand their biological activity. We can also use the computer to design molecules that can be used as drugs.

## COMPUTER SIMULATIONS BEYOND BIOLOGY—VISION FOR THE FUTURE

### Diversity, Diversity, Diversity

Biological systems face a unique challenge: they need to be prepared to deal with unexpected situations that might occur sometime in the future. How can any system be prepared for scenarios that have never been experienced before? The answer is simple: through diversity. Nature attempts to create a large range of variations within a system so that the system can adapt and modify its processes to deal with unforeseen challenges.

In animals, for example, each offspring receives a random half of the genetic information (DNA) of each of its parents, so each offspring is unique and enhances the diversity of the species. In this way, for a whole group of animals, the readiness to respond to possible future scenarios increases, and this increases the collective resilience of the species to unexpected situations.

I think that this diversity principle that biology teaches us is also applicable in many other aspects in life. For example, a strong society is a diverse society, in which different people, of different social backgrounds, sexes and educations, should learn to live together and understand and accept one another. Indeed, at school, or at home, we always have to find ways to negotiate with the people around us. Sometimes we have to deal with difficult and complex social situations, and of course some of us are better than others at resolving the conflicts we encounter. Furthermore, our life itself is diverse, with ups and downs and unexpected situations. The key for a better future and a stable society relies on our capabilities to handle life’s diversities successfully. Dealing with a diverse range of social and personal situations requires a well-developed **emotional intelligence**. I believe that we can use computer simulations to help us improve our emotional intelligence.

### Computer Simulations for the Development of Emotional Intelligence

**Figure 3:**
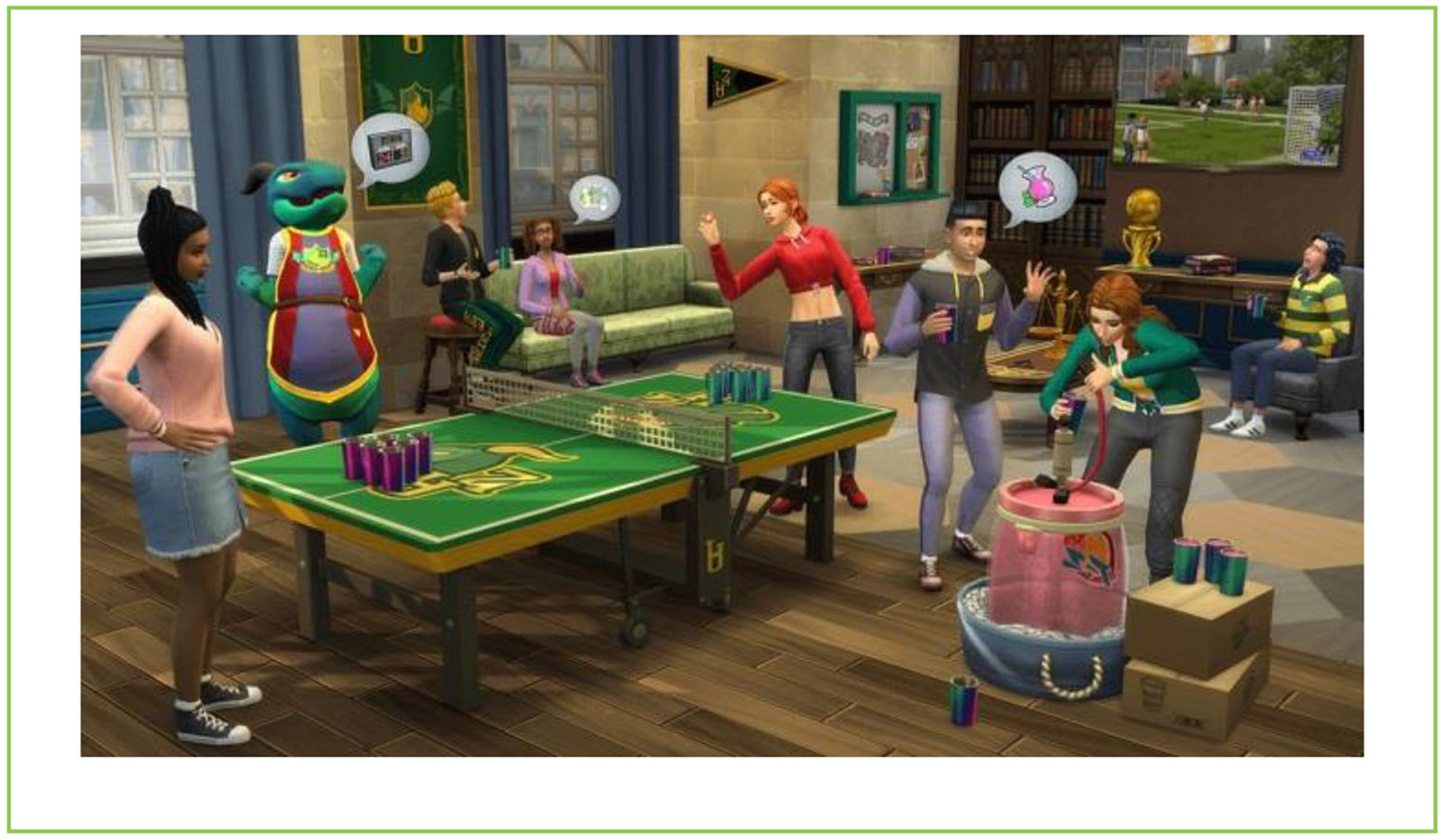
Computer simulation for developing emotional intelligence. Computer simulations may be able to teach us about complex situations in life. Imagine a game like the one shown, which allows you to experience a complex social situation and to try different ways of reacting and responding to the situation. Such a game could prepare you to better deal with real-life situations and with the diverse responses of different people: it could help you develop a more sophisticated emotional intelligence (Adopted from Rockpapershotgun).

A computer simulation to improve emotional intelligence could be in the form ofan interactive game that simulates a difficult social situation and allows you to pursue different strategies for resolving the problem (Figure 3). For example, someone insults you in class. How should you react to ensure that you do not totally destroy any possibility of working cooperatively with that person? Using a computer simulation, you could see the outcomes ofa diverse range of different actions that you might take. This type of activity, done both individually and as part of the educational system, could play an important role in enhancing the development of emotional intelligence.

## RECOMMENDATIONS FOR YOUNG MINDS

I want to share with you some insights that I gained from my scientific career and from life in general. First, it is important to do what you love. Do not do what your parents want you to do or what society tells you to do; try doing what you genuinely love doing. There is no better life than a life in which you do what you really love doing. Second, do not give up. Believe in yourself and do not get too excited by success or failure. Remember that every bad thing has something good in it, and every good thing has something bad in it, and we learn from both. Keep believing in yourself and eventually others will also believe in you. Third, try to be original. Each of us is special and unique. Try expressing your uniqueness and not just copy others. Fourth, be ready to make mistakes. I always say that a good scientist is someone who makes mistakes 90% of the time, and a really good scientist makes mistakes 99% of the time. Why? Because if you are excellent in your field, you deal with the most difficult problems. If you are not prepared to make mistakes, you will never deal with the more challenging things. Fifth, be a kind person—be generous and warm—these are important qualities to nurture.

The last thing I would like to recommend to you is related to planning. I think that in life, while you do need to be able to plan ahead, too much planning can lead to disappointment. Life never goes exactly the way we planned and surprising things often happen that are not part of the plan. If you are too busy with your original plan, you will not even notice new opportunities. The ideal is a delicate balance between following plans and being ready to respond to the surprises that life brings.

## YOUNG REVIEWERS

### NATAN ALTERMAN ORT JUNIOR HIGH SCHOOL, ISRAEL, AGES: 13–15

Technological Scientific classes in “Beit Chinuch” are classes containing excellent students in the fields of science and technology. The students are curious about everything relating to science, always questioning the world around them in order to understand it better.

## Abbreviations

COMPUTER SIMULATION: A tool for performing scientific studies based on computations using a computer. You can think of computer simulations like computer games that help scientists to learn and better understand the phenomena they investigate.
PROTEINS: Big biological molecules that perform all the functions of life—build the body, take part in chemical reactions, digest food, etc. You can think of proteins as necklaces, composed of different types of beads, that fold into unique 3D shapes, so that each protein has its own unique folded shape.
STRUCTURAL BIOLOGY: A research field that studies the structure of large molecules (macromolecules) built from collections of smaller molecules. Researchers try to understand the principles by which molecules fold to create a certain 3D structure.
EMOTIONAL INTELLIGENCE: The ability to identify emotions in yourself and others, understand them, and use them to interact better with different people.
